# ^68^Ga-PSMA-HBED-CC PET imaging in breast carcinoma patients

**DOI:** 10.1007/s00259-016-3563-6

**Published:** 2016-11-08

**Authors:** Mike Sathekge, Thabo Lengana, Moshe Modiselle, Mariza Vorster, JanRijn Zeevaart, Alex Maes, Thomas Ebenhan, Christophe Van de Wiele

**Affiliations:** 1 Department of Nuclear Medicine, University of Pretoria and Steve Biko Academic Hospital, Private Bag X169, Pretoria, 0001 South Africa; 2 Department of Nuclear Medicine, AZ Groeninge, Kortrijk, Belgium; 3 Department of Radiology and Nuclear Medicine, University Ghent, Ghent, Belgium

**Keywords:** ^68^Ga-PSMA, PET/CT, Breast cancer

## Abstract

**Background:**

To report on imaging findings using ^68^Ga-PSMA-HBED-CC PET in a series of 19 breast carcinoma patients.

**Methods:**

^68^Ga-PSMA-HBED-CC PET imaging results obtained were compared to routinely performed staging examinations and analyzed as to lesion location and progesterone receptor status.

**Results:**

Out of 81 tumor lesions identified, 84% were identified on ^68^Ga-PSMA-HBED-CC PET. ^68^Ga-PSMA-HBED-CC SUVmean values of distant metastases proved significantly higher (mean, 6.86, SD, 5.68) when compared to those of primary or local recurrences (mean, 2.45, SD, 2.55, *p* = 0.04) or involved lymph nodes (mean, 3.18, SD, 1.79, *p* = 0.011). SUVmean values of progesterone receptor-positive lesions proved not significantly different from progesterone receptor-negative lesions. SUV values derived from FDG PET/CT, available in seven patients, and ^68^Ga-PSMA-HBED-CC PET/CT imaging proved weakly correlated (*r* = 0.407, *p* = 0.015).

**Conclusions:**

^68^Ga-PSMA-HBED-CC PET/CT imaging in breast carcinoma confirms the reported considerable variation of PSMA expression on human solid tumors using immunohistochemistry.

## Introduction

Prostate-specific membrane antigen (PSMA) is an integral membrane protein, mapped to chromosome 11q14, which is over-expressed by a high number of prostate carcinomas; this expression is further increased in higher-grade carcinomas, in metastatic disease, and in hormone refractory prostate carcinomas, making it an interesting target for prostate carcinoma-specific imaging and therapy [1]. In this regard, the PSMA inhibitor Glu-NH-CO-NH-Lys(Ahx)-HBED-CC was labeled with ^68^Ga for positron emission tomography (PET) and shown to be more accurate for the detection of recurrent prostate carcinoma when compared to ^18^F-choline PET and, in combination with MRI, to be significantly more accurate for the detection of primary prostate carcinoma when compared to PET/CT [2–4]. Aside from prostate carcinoma, PSMA has also been reported to be selectively overexpressed in the tumor-associated neovasculature of a wide variety of solid tumors including breast carcinoma [5–8].

Sathekge et al. recently presented the first case of a patient with metastatic breast cancer, in whom PET/CT using the Glu-NH-CO-NH-Lys-(Ahx)-[^68^Ga(HBEDCC)] (^68^Ga-PSMA) ligand detected bone and liver metastases with essentially similar visual contrast to ^18^F-FDG PET/CT [6]. In this study, we built on these initial findings by reporting on imaging findings using ^68^Ga-PSMA-HBED-CC PET in a series of 19 breast carcinoma patients.

## Patients and methods

Nineteen women (mean age, 45 years, range, 25-66 years) suffering from breast carcinoma were prospectively included in this study, approved by the Institutional Ethics Committee, following written informed consent. ^68^Ga-PSMA-HBED-CC PET imaging was performed in nine “de novo” diagnosed breast carcinoma patients, in five patients presenting with a loco-regional recurrence of breast carcinoma, and in a pre-treatment metastasized setting in another five patients. Six patients were progesterone receptor-positive and seven were progesterone receptor-negative. In the remaining six patients, progesterone receptor status was unknown. Seven of the 19 patients included additionally underwent FDG PET/CT imaging (three de novo patients, two loco-regional recurrent, and two metastasized patients). Both ^68^Ga-PSMA-HBED-CC and FDG PET/CT imaging was performed from the top of the pelvis to the skull following the injection of a body weight-adjusted dose, ((body weight/10) + 1) × 37 MBq for FDG PET imaging and 2 MBq/kg for ^68^Ga-PSMA-HBED-CC PET imaging. All ^68^Ga-PSMA-HBED-CC injections contained 2 mmol PSMA ligand, resulting in a median specific radioactivity of 66 GBq/µmol [9]. In all patients, available imaging data performed as part of the staging or restaging procedure, including contrast-enhanced CT imaging of the thoraco-abdominal region, echography, bone scintigraphy, and, when available, FDG-PET imaging (see also above, performed within 2 weeks from the ^68^Ga-PSMA-HBED-CC PET examination and prior to any treatment initiation), were used as gold standard to define the imaging potential of ^68^Ga-PSMA-HBED-CC PET imaging.

## Statistical analysis

Differences in ^68^Ga-PSMA-HBED-CC SUVmean values between different subgroups were assessed using Student’s *t* test or ANOVA with post hoc Bonferroni correction where appropriate. Correlation analysis was performed using Pearson’s correlation or Spearman-rank correlation analysis where appropriate.

## Results

**Fig. 1:**
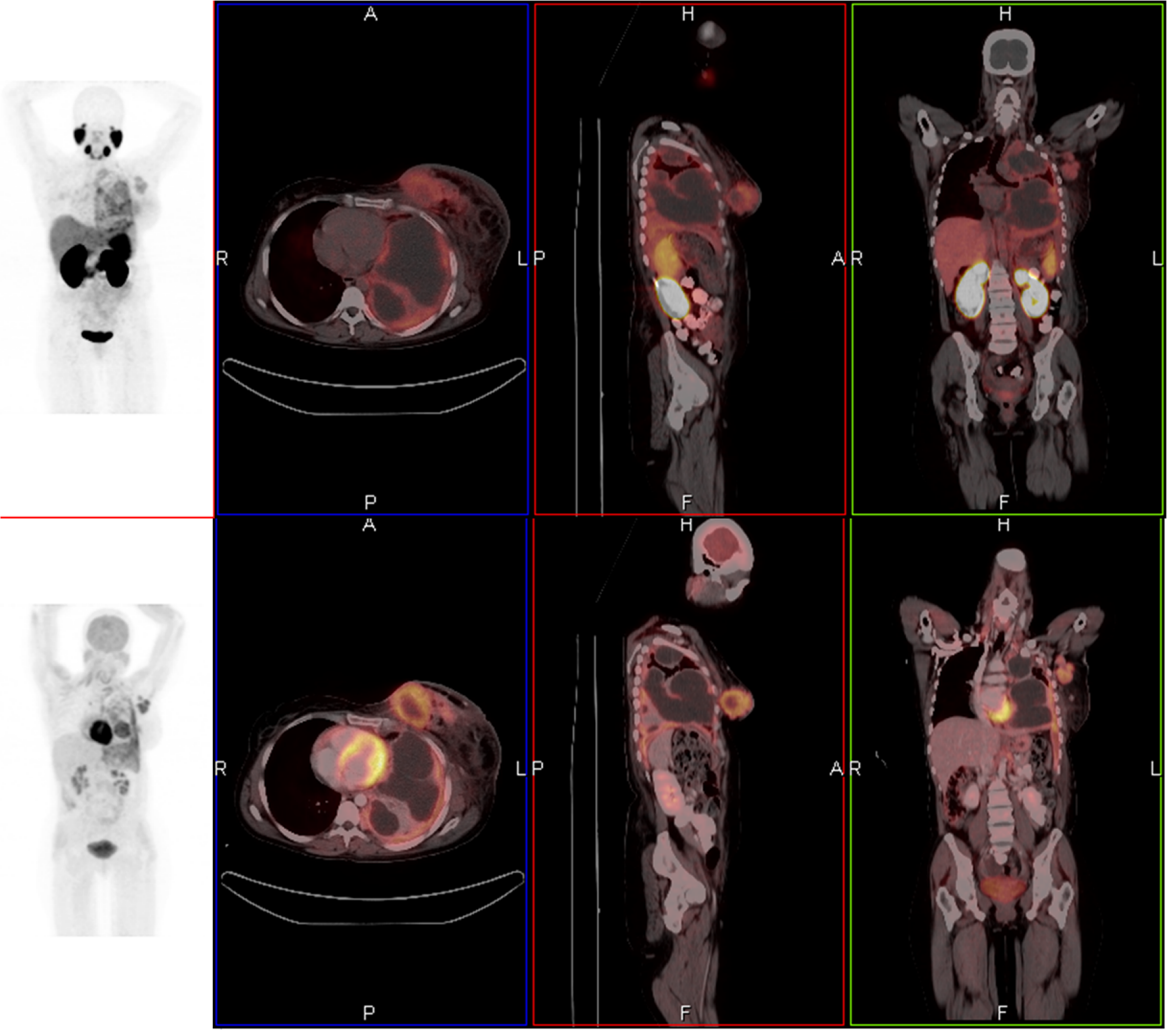
A 42-year-old female with metastatic breast carcinoma who underwent ^68^Ga-PSMA and ^18^F-FDG PET/CT. Axial, coronal, and sagittal fused ^68^Ga-PSMA PET/CT images demonstrated primary left breast cancer, axillary nodal and left pleural metastases (**a**). Avidity is slightly intense on ^18^F-FDG PET/CT images (**b**). Maximum-intensity-projection PET gives overview of all lesions (**c**, **d**)

**Fig. 2:**
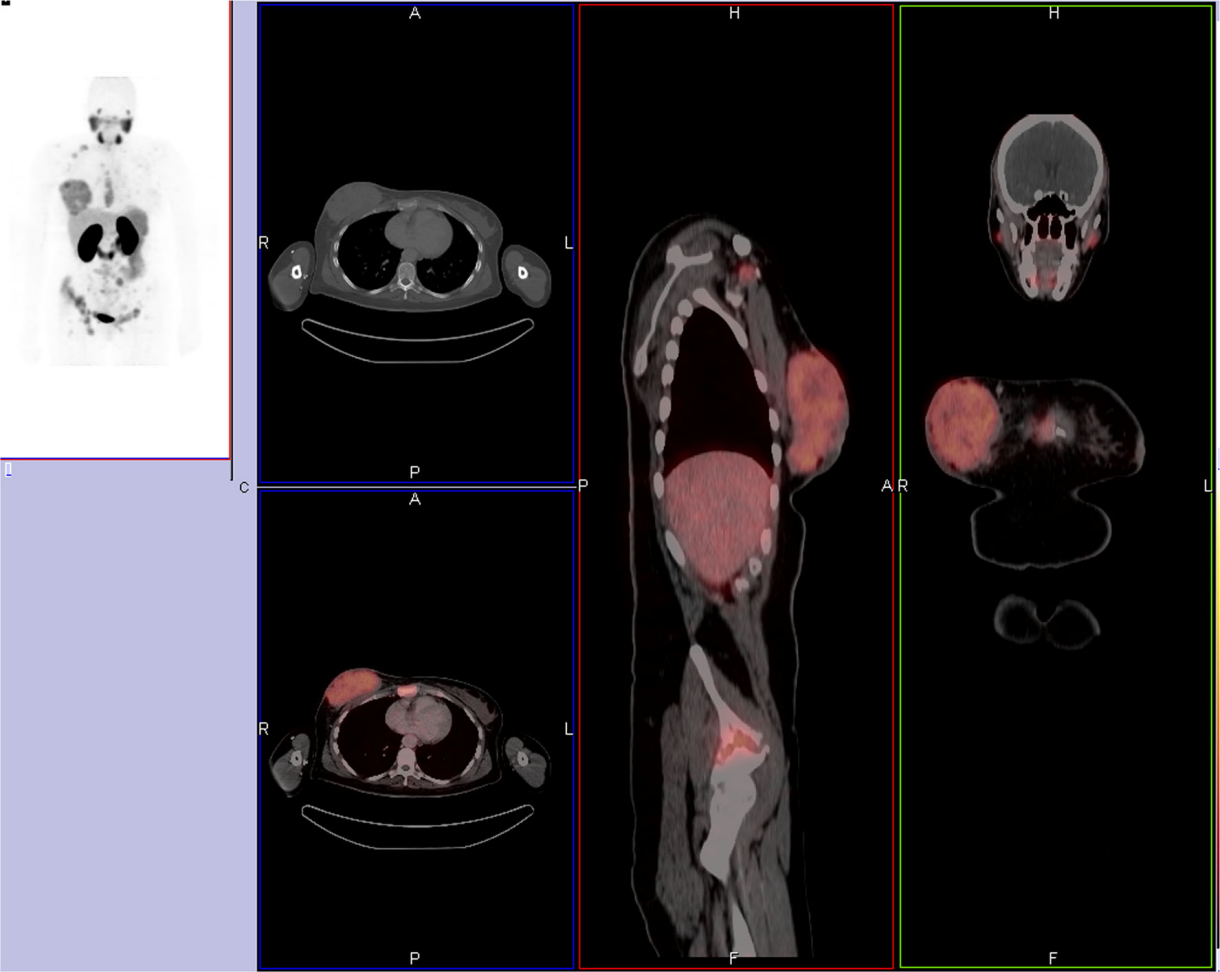
A 39-year-old woman with stage IV by ^68^Ga-PSMA PET/CT. **a** Maximum-intensity-projection PET demonstrated multiple osseous metastasis and a primary right breast cancer. Axial, coronal, and sagittal fused PET/CT confirms all the lesions (**b**)

Overall, in the 19 patients studied, 81 tumor lesions were identified: 13 primary tumors and/or local recurrences, 15 involving the lymph nodes, and 53 metastases (see Table 1 and Figs. 1 and 2). Out of these, six primary or recurrent lesions, two lymph nodes, and five metastases proved negative on ^68^Ga-PSMA-HBED-CC PET, yielding an overall detection rate of 84% for ^68^Ga-PSMA-HBED-CC PET.

**Table 1:** Patient characteristics and PSMA imaging results (lesions identified on ^68^Ga-PSMA-HBED-CC PET/total number derived from routine examination procedures)

Patient no.	Age	Carcinoma type	PR status	Clinical setting	Primary/local relapse	LN	M+
1	45	Ductal	NA	Primary	1/1	0/0	0/0
2	45	NA	NA	M+	0/0	0/0	5/5
3	49	Ductal	NA	M+	0/0	0/0	4/6
4	66	Lobular	PR+	Primary	0/1	1/1	0/0
5	40	NA	NA	Recurrence	0/1	1/1	4/4
6	39	Ductal	PR+	Recurrence	1/1	0/0	0/1
7	38	Ductal	PR+	Recurrence	0/1	0/0	4/5
8	39	Ductal	PR–	Primary	0/1	0/0	0/1
9	25	Ductal	PR–	Primary	1/1	1/1	0/0
10	62	Ductal	PR–	Recurrence	0/1	0/0	0/0
11	53	Lobular	PR+	Primary	1/1	2/2	4/4
12	54	Neuroendocrine differentiation	PR+	Primary	1/1	1/2	5/5
13	42	Ductal	PR+	Primary	1/1	1/1	4/4
14	39	Ductal	PR–	Primary	1/1	1/2	2/3
15	57	Ductal	NA	Primary	0/1	0/0	0/0
16	40	Ductal	NA	M+	0/0	2/2	4/4
17	31	Ductal	PR–	M+	0/0	0/0	4/4
18	44	NA	PR–	Recurrence	0/0	2/2	5/5
19	56	Ductal	PR–	M+	0/0	1/1	2/2

*NA* not available, *PR* progesterone receptor, *M+* metastasized

^68^Ga-PSMA-HBED-CC SUVmean values of distant metastases proved significantly higher (mean, 6.86, SD, 5.68) when compared to those of primary or local recurrences (mean, 2.45, SD, 2.55, *p* = 0.04) or involved lymph nodes (mean, 3.18, SD, 1.79, *p* = 0.011).

^68^Ga-PSMA-HBED-CC SUVmean values of progesterone receptor-positive lesions (*n*, number of lesions = 31) proved not significantly different from those obtained in progesterone receptor-negative lesions (*n* = 31), respectively 5.62 ± 5.40 (mean/SD) versus 4.19 ± 2.63 (*p* = 0.188).

FDG PET/CT imaging performed in seven patients identified 35 lesions. Of the 35 FDG-positive lesions, six proved PSMA-negative and FDG PET/CT was clearly more intense than ^68^Ga-PSMA with regards to primary lesions (Fig. 1). Inversely, one lesion identified on ^68^Ga-PSMA-HBED-CC PET proved FDG PET/CT-negative. In those patients that underwent both examinations,^68^Ga-PSMA values proved not significantly different from those obtained using FDG [mean 4.58, SD, 3.94) versus 6.1 (SD, 2.82), *p* = 0104].

Of interest, a weak but significant relationship was identified between SUV values derived from FDG PET/CT (mean, 6.1, SD, 2.82) and ^68^Ga-PSMA-HBED-CC PET/CT imaging (*r* = 0.407, *p* = 0.015).

## Discussion

PSMA has been previously shown to be universally up-regulated on tumor-associated vascular endothelial cells in solid tumors and to participate in matrix degradation and facilitate integrin signaling and p21-activated kinase 1 (PAK-1) activation leading to productive tumor invasion [10]. Since PSMA is found in the neovasculature of many tumors, it is thought to regulate angiogenesis, however, the precise mechanism by which PSMA exerts its effect is unknown [10, 11]. To this effect some groups suggest that PSMA plays a number of roles in angiogenesis, some involving vascular endothelial factor (VEGF), others not [11, 12]. In a study by Wernicke et al. on breast carcinoma patients, tumor-associated vasculature was shown to be PSMA-positive in 68 out of 92 primary breast cancers (74%) and in 14 out of 14 of breast cancers metastatic to the brain [7]. Likewise, in a study by Natsuko et al., five breast cancer brain metastases showed PSMA expression on tumor blood vessels [8], and recently our manuscript demonstrated intense uptake by ^68^Ga-PSMA-HBED-CC in metastatic breast cancer [6]. In line with these findings, out of 81 tumor lesions identified, 84% were proven to be ^68^Ga-PSMA PET-positive in the series that presented with distant metastases displaying significantly higher ^68^Ga-PSMA-HBED-CC SUV values. Furthermore, ^68^Ga-PSMA-HBED-CC SUV values of tumor lesions were shown to vary significantly from one patient to another as well as from one lesion to another within one patient. These findings concur with the reported considerable variation of PSMA expression on human solid tumors using immunohistochemistry, thus further supporting the fact that breast cancer is a heterogeneous disease [13].

The hormonal receptor (estradiol receptor (ER)/progesterone receptor (PR) status is a strong prognostic factor for breast cancer. The progesterone receptor (PR) is an estrogen response element that is transcribed after effective binding of the estradiol-estradiol receptor (ER) complex to DNA in ER-positive, estradiol-responsive breast cancers [14]. In the study by Wernicke et al., patients with PR-negative tumors were more likely to present with a more extensive PSMA staining (PSMA-expression in > 50% of microvessels) when compared to PR-positive tumors [7]. In our series presented, no significant difference in ^68^Ga-PSMA SUV values between PR-positive and PR-negative tumors could be identified. However, in some patients under study, a considerable time interval existed between characterization of the PR-status on the primary tumor and subsequent imaging, performed in a metastasized setting. Accordingly, the tumor biology of some of these tumors may have changed due to ongoing mutations resulting in a loss of PR expression, thereby flawing the existence of a possible relationship between both variables.

Furthermore, there is increasing evidence of temporal and spatial heterogeneity in breast cancer receptor overexpression. Patients with negative test results at diagnosis can have positive test results later in the disease course and vice versa, a fact that explains why biopsy of metastatic disease is a strong recommendation of many clinical treatment guidelines [13]. Hence, heterogeneity in biomarker expression at metastatic sites is only beginning to be recognized, with growing appreciation for molecular imaging.

Since the use of ^18^F-FDG tumor uptake as a biomarker for predicting a pathologic response to treatment has been explored in the preclinical and clinical settings, with conflicting results [15], we also needed to demonstrate the role of ^18^F-FDG in advanced disease. More so, limited evidence supports the use of ^18^F-FDG PET to evaluate the extent of disease in selected patients with recurrent or metastatic disease [16, 17].

Although our case demonstrated concordance of ^68^Ga-PSMA and ^18^F-FDG lesions [6], of interest, we identified a weak but significant relationship between tumor metabolism as assessed by FDG uptake and tumor angiogenesis assessed by ^68^Ga-PSMA-HBED-CC PET imaging. This finding is in line with a previous report by Grobes et al. in a series of 20 consecutive newly diagnosed breast carcinoma patients in whom FDG uptake proved significantly associated with the degree of angiogenesis assessed using immunohistochemistry and CD105 staining [1, 18]. CD105 or endoglin is an accessory receptor for transforming growth factor beta (TGF-beta) of which the expression is up-regulated in actively proliferating endothelial cells. Most investigators, including Grobes et al., have reported a correlation between tumor angiogenesis and glucose metabolism [12, 19]. However, other studies failed to demonstrate a significant correlation between angiogenesis and FDG uptake. Avril et al. reported an inverse relationship between SUV and the number of microvessels in breast cancer patients [20]. This could be one of the reasons for the weak relationship between FDG uptake and ^68^Ga-PSMA-HBED-CC PET imaging.

The robust expression of PSMA by breast cancer lesions as evidenced using ^68^Ga-PSMA-HBED-CC PET imaging in this series and the absence of PSMA on normal vascular endothelium as well as its limited expression on the luminal side of the intestinal epithelium, which is not accessible via the vasculature, makes PSMA an interesting potential target for antiangiogenic therapy of breast carcinoma. More specifically, PSMA-targeting therapeutic agents may selectively destroy vessels perfusing tumor tissue and achieve high regional doses of drugs to overcome tumor resistance while sparing normal tissue, which typically lacks PSMA expression. In this regard, both the anti-PSMA monoclonal antibody J591 and 177Lu-PSMA-617 were shown to be well tolerated and to show considerable clinical efficacy, respectively in patients suffering from a variety of advanced solid tumors and prostate carcinoma [21, 22]. More recently, the results of a first-in human phase I trial to determine the safety, pharmacokinetics, and anti-tumor activity of BIND-014, a PSMA-targeting nanoparticle containing docetaxel were reported [23]. BIND-014 was shown to be generally well tolerated and clinical activity was noted in multiple tumor types.

Folkman characterized angiogenesis as being fundamental for tumor growth beyond 2 mm in 1971 [24]. Surprisingly, there is still no validated predictive biomarker for the selection of antiangiogenic therapy [25]. While angiogenesis is an important component in the progression of a number of diseases, it is clear that all angiogenic processes are not regulated by the same signals and are often distinct pathologies [26]. Hence ^68^Ga-PSMA-HBED-CC PET imaging as performed in the series presented may allow for selection of those patients most likely to benefit from these PSMA-targeting treatment modalities. Furthermore, it is not to be excluded that ^68^Ga-PSMA-HBED-CC PET imaging may also play a role in treatment response monitoring and selection of those patients suffering from breast carcinoma that may benefit from non-PSMA targeting antiangiogenic treatment strategies either given as monotherapy or in combination with chemotherapy, e.g., bevacizumab, aflibercept, integrin targeting antibodies, sunitinib, sorafenib, gamma-secretase inhibitors, angiopoietin inhibitors, and mTOR inhibitors [27].

The limitations of this study were the small number of patients included and lack of assessment of HER2 status of the metastatic lesions. This will be undertaken in a future large study. Although our study did not assess targeting antiangiogenic therapy for breast cancer; studies assessing the potential of ^68^Ga-PSAM-HBED-CC for predicting and monitoring response to antiangiogenic treatment in patients suffering from breast carcinoma could be helpful and thus warranted. In conclusion, ^68^Ga-PSMA-HBED-CC PET/CT imaging in breast carcinoma confirms the reported considerable variation of PSMA expression on human solid tumors using immunohistochemistry.
